# Prevalence of non-Hodgkin lymphoma patients at high-risk of failure after CAR T-cell therapy eligible for bridging radiation therapy

**DOI:** 10.3389/fonc.2024.1425506

**Published:** 2024-08-19

**Authors:** Adnan Danish, Alexandra Della Pia, Lindsay Fogel, Hassan Alkhatatneh, Charles Zhao, Tony Varughese, Karine A. Al Feghali, Lauren Pascual, Brittany Sinclaire, Michael Marafelias, Joshua Zenreich, Yen-Hong Kuo, Tatyana A. Feldman, Yi Zhang, Andre H. Goy, Andrew Ip, Scott D. Rowley

**Affiliations:** ^1^ John Theurer Cancer Center, Hackensack Meridian Health, Hackensack, NJ, United States; ^2^ Lymphoma Division, Hackensack University Medical Center, Hackensack, NJ, United States; ^3^ Department of Medical Sciences, Hackensack Meridian School of Medicine, Nutley, NJ, United States; ^4^ Department of Medicine, Englewood Hospital and Medical Center, Englewood, NJ, United States; ^5^ RefleXion Medical, Inc., Hayward, CA, United States; ^6^ Office of Research Administration, Hackensack Meridian Health Research Institute, Nutley, NJ, United States; ^7^ Center for Discovery and Innovation, Hackensack Meridian Health, Hackensack, NJ, United States; ^8^ Department of Oncology, Georgetown University School of Medicine, Washington, DC, United States

**Keywords:** CD19 CAR T-cell therapy, relapsed or refractory non-Hodgkin lymphoma (R/R NHL), bridging radiotherapy, bridging therapy, radiotherapy

## Abstract

**Background and purpose:**

The aim of this study was to determine the prevalence of patients with relapsed or refractory (R/R) non-Hodgkin lymphoma (NHL) meeting high-risk criteria for early relapse after CD19 CAR T-cell therapy (CART) who have disease encompassable in a standard radiation therapy (RT) plan (defined as <5 malignant lesions) and may benefit from bridging RT prior to CD19 CART.

**Materials and methods:**

This is a single-center, retrospective study of patients with R/R NHL who received CD19 CART from 2018 to 2022. Eligible patients had pre-apheresis radiologic studies available. All patients were classified by number of lesions and history of high-risk disease criteria: bulky disease ≥10 cm, ≥1 extranodal (EN) sites, LDH ≥normal, or ≥1 lesion with SUVmax ≥10.

**Results:**

A total of 81 patients with R/R NHL were evaluated. Based on our definition, 40 (49%) patients would have been eligible for bridging RT, including 38 patients who met high-risk criteria: 31 with ≥1 EN site, 19 had ≥1 lesion with SUVmax ≥10, 16 with bulky disease, and 3 with elevated LDH. At 3 months after CART, ORRs in high-risk patients with <5 lesions, ≥5 lesions, and no lesions on pre-apheresis studies were 76% (CR 69%, PR 7%), 70% (CR 60%, PR 10%), and 80% (CR 80%), respectively.

**Conclusion:**

Approximately 47% (38/81) of patients were classified as at high risk of relapse after CART with disease encompassable in a standard radiation plan and eligible for bridging RT studies.

## 1 Introduction

Non-Hodgkin lymphoma (NHL) is the most common hematologic malignancy in the United States, representing 4% to 5% of all new cancers (1). Although the majority of patients respond to first-line immunochemotherapy regimens such as rituximab, cyclophosphamide, vincristine, doxorubicin, and prednisone (R-CHOP), approximately 30% to 40% of individuals relapse and require additional therapies (2, 3). The risk of relapse is predicted by NHL subgroup and by disease risk stratification for both aggressive and indolent lymphomas that include such factors as disease bulk, LDH elevation, and extranodal disease (4, 5). The development of CD19-directed chimeric antigen receptor T-cell therapy (CD19 CART) altered the treatment landscape for patients with relapsed or refractory (R/R) B-cell NHLs as it provides a new option for patients who previously had limited other effective alternatives (2). Despite its remarkable success for patients with R/R B-cell NHL, however, longer follow-up of the multicenter ZUMA-1 and JULIET trials for axicabtagene ciloleucel and tisagenlecleucel, respectively, demonstrated that more than half of patients experience disease recurrence (6). Given this high rate of relapse associated with poor survival outcomes, it is imperative to explore new strategies for preventing relapse among patients with R/R B-cell NHL who receive CD19 CART.

Prior studies identified several disease features at the time of CART treatment that predict increased risk of early progression following CD19 CART in patients with B-cell NHL. These high-risk factors include an Eastern Cooperative Oncology Group Performance Status (ECOG PS) ≥2, elevated lactate dehydrogenase (LDH) levels, disease involvement of ≥2 extranodal (EN) sites, and, particularly, bulky tumor mass (7). Another retrospective study in patients with R/R large B-cell lymphoma similarly identified predictors of early CD19 CART failure, which included the presence of either bulky lesions (defined as ≥5 cm in diameter) or necrotic tissue in tumor lesions (6).

Bridging therapy, defined as treatment of disease during the obligate manufacturing period after collection of lymphocytes and before the CART product is released for infusion, may help control bulky, high-volume, extranodal, or rapidly progressing disease. Multiple reports (mostly with limited numbers of subjects) describe a potential improvement in post-CART survival with the use of palliative bridging radiation therapy (RT) to persistent sites of disease before CART (8–12). Furthermore, in addition to this purely palliative effect, there is developing evidence that radiotherapy may alter the tumor microenvironment (TME) with a direct immunomodulatory effect leading to improved CART response. Radiation therapy can result in either immunostimulatory or immunosuppressive TME effects, depending on the dose fractionation and total dose administered (13, 14). However, the proportion of patients who may benefit from bridging RT is unknown, hindering the development of prospective clinical studies exploring the biology of RT in the context of CART. In this study, we aim to determine the prevalence of patients with NHL meeting high-risk criteria who have disease that could be encompassed in a standard radiation plan prior to receiving CD19 CART.

## 2 Materials and methods

### 2.1 Study design

This is a single-center, retrospective study of patients with R/R B-cell NHL who received CD19 CART at Hackensack University Medical Center from March 2018 to July 2022. This study was reviewed and approved by the Institutional Review Board (IRB) at Hackensack Meridian Health (Pro2021-0256). This trial was conducted under the International Conference on Harmonization Good Clinical Practice guidelines and according to the ethical principles from the Declaration of Helsinki.

### 2.2 Eligibility criteria

Patients were eligible for inclusion in this study if they were at least 18 years of age or older, had a diagnosis R/R B-cell NHL, and received a commercial CD19 CART product between March 2018 and July 2022 (*N* = 81). Eligible patients were those with pre-apheresis radiologic studies [e.g., positron emission tomography (PET) scan or computed tomography (CT) scan] available for review (**Supplementary Figure 1**). Pre-apheresis imaging studies obtained at a median of 48 days (range, 7–236 days) before apheresis were used to identify those patients with oligometastic disease (OMD) encompassable in a standard radiation plan defined for this study as <5 distinct malignant lesions (15). Total metabolic volume was not calculated for the purposes of this study. Consultation with a radiation oncologist was not required for study enrollment.

### 2.3 Data collection

Data were obtained from Hackensack Meridian Health’s electronic health record and manual chart review using the date of CD19 CART infusion for calculation of post-treatment survival. Demographic data included age, sex, Karnofsky performance status at the time of CART, and disease type and specific CD19 CART product administered (e.g., axicabtagene ciloleucel, tisagenlecleucel, lisocabtagene maraleucel, or brexucabtagene autoleucel). Disease staging at the time of diagnosis included number of extranodal sites, presence of bulky disease, and LDH value. Patients were stratified as being at high risk of relapse if they had one or more of the following characteristics: bulky disease, ≥1 site of EN disease, elevated LDH, and/or ≥1 lesion with maximum standard uptake value (SUVmax) ≥10. Bulky disease was defined as any lesion measuring ≥10 cm in diameter. Elevated LDH was defined as a value greater than the upper limit of the lab normal range (LNR). Treatment history included the number of prior lines of therapy and number and SUVmax of lesions on pre-apheresis imaging. Treatment response was determined on restaging studies at 3 months after CD19 CART infusion. The dataset was reviewed, and uncertain data were adjudicated by the primary physicians (AD and AI) overseeing data collection and conduct of the study.

### 2.4 Outcomes

The primary outcome was to determine the proportion of patients with NHL who have OMD that could be encompassed in a standard radiation plan immediately before CD19 CART infusion and would, therefore, be eligible for bridging RT. Secondary outcomes included the prevalence of high-risk features by type, clinical response at 3 months after CD19 CART infusion, overall survival (OS), progression-free survival (PFS), and outcomes of patients who received bridging RT prior to CD19 CART (if administered). Clinical response was classified as the overall response rate (ORR) including complete response (CR) or partial response (PR) as defined using the Cheson Criteria 2014 based on radiologic studies in combination with the treating physician’s documentation (16). OS was defined as the number of months from the date of CART infusion to the date of death or censoring at the time the dataset was closed for analysis in July 2022. PFS was defined as the number of months from the date of CD19 CART infusion to the date of first event (disease progression or death) or censoring at the time the dataset was closed for analysis in July 2022. The median follow-up of patients included in this study is 9.5 months (interquartile range, 6–18 months).

### 2.5 Statistical analysis

The numerical variables were summarized using the median, interquartile range, and range. The categorical variables were summarized using frequencies and percentage. For the duration of survival time, the Kaplan–Meier procedure was used to estimate the median time, and the standard error was estimated using the Greenwood’s formula. The Kaplan–Meier curves were generated. The log-rank test was used to compare the time (Kaplan–Meier curves) between groups. The two-sided *p*-value was reported for each test. A *p*-value less than 0.05 was considered an indication of statistical significance. Statistical analysis was performed using the R language (17).

## 3 Results

There were 81 patients with R/R B-cell NHL who had pre-apheresis radiologic studies available and received a CD19 CART product at our institution between March 2018 to July 2022. The median age was 65 years (range, 23–84), 60% were male, 43% had a Karnofsky performance status of ≥80, and 43% had a Ki-67 proliferation index ≥80% (**Table 1**). Lymphoma types included a predominance of diffuse large B-cell lymphoma (75%) followed by follicular lymphoma (11%), mantle cell lymphoma (7%), primary mediastinal B-cell lymphoma (5%), and chronic lymphocytic leukemia (1%). Patients received a median of 2 (range, 1–7) prior lines of therapy, including autologous (16%) and allogeneic (5%) hematopoietic stem cell transplantation. CD19 CART products administered included axicabtagene ciloleucel (62%), lisocabtagene maraleucel (16%), tisagenlecleucel (15%), and brexucabtagene autoleucel (7%).

**Table 1:** Patient characteristics.

Characteristic	Patients *N* = 81 *n* (%)
Age, years, median (interquartile range)	65 (57–73)
Sex Male Female	49 (60) 32 (40)
Disease type Chronic lymphocytic leukemia (CLL) Diffuse large B-cell lymphoma (DLBCL) Follicular lymphoma Mantle cell lymphoma Primary mediastinal B-cell lymphoma	1 (1) 61 (75) 9 (11) 6 (7) 4 (5)
Karnofsky performance status ≥80 60–70 Unknown	42 (52) 37 (46) 2 (2)
Ki-67 proliferation index ≥80% >30% to <80% ≤30% Unknown	35 (43) 28 (35) 12 (15) 6 (7)
Prior lines of therapy, median (interquartile range)	2 (2–3)
Previous stem cell transplant Autologous Allogeneic	13 (16) 4 (5)
Primary refractory	35 (43)
CD19 CAR T-cell product infused Axaxicabtagene ciloleucel Brexucabtagene autoleucel Lisocabtagene maraleucel Tisagenlecleucel	50 (62) 6 (7) 13 (16) 12 (15)

We could not correlate OMD or polymetastatic disease (PMD, defined as ≥5 sites on pre-apheresis radiologic studies) with the presence of high-risk disease features. Forty (49%) patients met our criteria for OMD (**Supplementary Figure 1**). Of these 40 patients, 38 (95%) presented with at least one high-risk criteria: 31 (78%) had ≥1 extranodal sites of disease, 19 (48%) had ≥1 lesion with SUVmax ≥10, 16 (40%) had bulky disease ≥10 cm, and 3 (8%) had LDH above the LNR (**Table 2**).

**Table 2:** High-risk features in patients with pre-apheresis radiologic studies.

Characteristic	Patients with <5 lesions *N* = 40 *n* (%)	Patients with ≥5 lesions *N* = 36 *n* (%)	Patients with no lesions *N* = 5 *n* (%)
High-risk criteria met*	38 (95)	36 (100)	5 (100)
Bulky disease ≥10 cm Yes No	16 (40) 24 (60)	18 (50) 18 (50)	3 (60) 2 (40)
≥1 extranodal sites Yes No	31 (78) 9 (22)	28 (78) 8 (22)	0 5 (100)
LDH ≥lab normal range Yes No	3 (8) 37 (92)	21 (58) 15 (42)	4 (80) 1 (20)
≥1 lesion with SUVmax ≥10 Yes No	19 (48) 21 (52)	28 (78) 8 (22)	0 5 (100)

*High-risk of progression defined as ≥1 of the following characteristics: bulky disease, ≥1 site of extranodal (EN) disease, elevated lactate dehydrogenase, and/or ≥1 lesion with maximum standard uptake value (SUVmax) ≥10.

Patients are stratified by the number of lesions on pre-apheresis radiologic studies into the following cohort: disease encompassable in a standard radiation plan (defined as <5 lesions on imaging) or disease not considered encompassable in a standard radiation plan (defined as no lesion or five or more lesions on imaging).

Of the remaining 41 patients, 36 had PMD and 5 were in CR on pre-apheresis imaging (**Supplementary Figure 1**). All of the 36 patients with ≥5 sites on pre-apheresis radiologic studies met at least one high-risk criteria: 28 (78%) had ≥1 extranodal sites of disease, 28 (78%) had ≥1 lesion with SUVmax ≥10, 21 (58%) had LDH above the LNR, and 18 (50%) had bulky disease ≥10 cm (**Table 2**). Of the five patients in CR at time of CD19 CART, all (100%) met at least one defined high-risk criteria at the time of diagnosis: four (80%) had LDH above the LNR and three (60%) had a history of bulky disease ≥10 cm (**Table 2**).

The clinical response data after CD19 CART are presented in **Table 3**. At 3 months, the ORR was 76% (CR 69%; PR 7%) for 29 high-risk patients with disease encompassable in a standard radiation plan. In patients with high-risk features but with more than five lesions on pre-apheresis studies, the 3-month ORR was 70% (CR 60%; PR 10%). In patients in CR on pre-apheresis imaging, the 3-month ORR was 80% (CR 80%).

**Table 3:** Clinical response at 3 months after CD19 CAR T-cell therapy stratified by the presence of high-risk criteria in patients with NHL.

Response	Patients with <5 lesions *N* = 40		Patients with ≥5 lesions High risk *N* = 20 *n* (%)	Patients with no lesions High risk *N* = 5 *n* (%)
	High risk *N* = 29 *n* (%)	Standard risk *N* = 1 *n* (%)		
**ORR**	22 (76)	–	14 (70)	4 (80)
**CR**	20 (69)	–	12 (60)	4 (80)
**PR**	2 (7)	–	2 (10)	–
**SD**	4 (14)	–	1 (5)	–
**PD**	3 (10)	1 (100)	5 (25)	1 (20)
**Unevaluable**	–	–	1	–
**Unknown**	9	1	15	–

**Figure 1:**
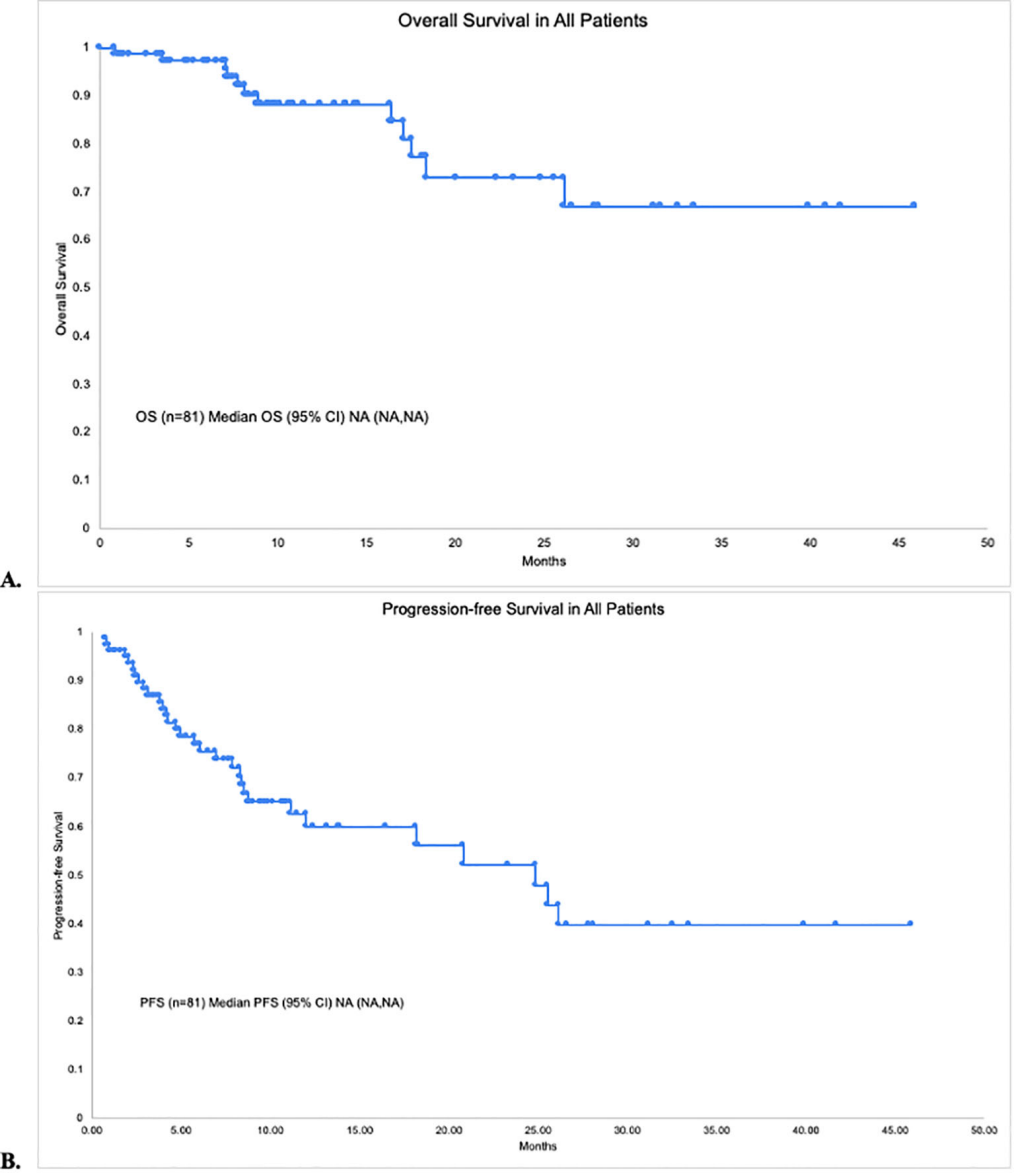
Survival in all patients. **(A)** Overall survival. **(B)** Progression-free survival.

**Figure 2:**
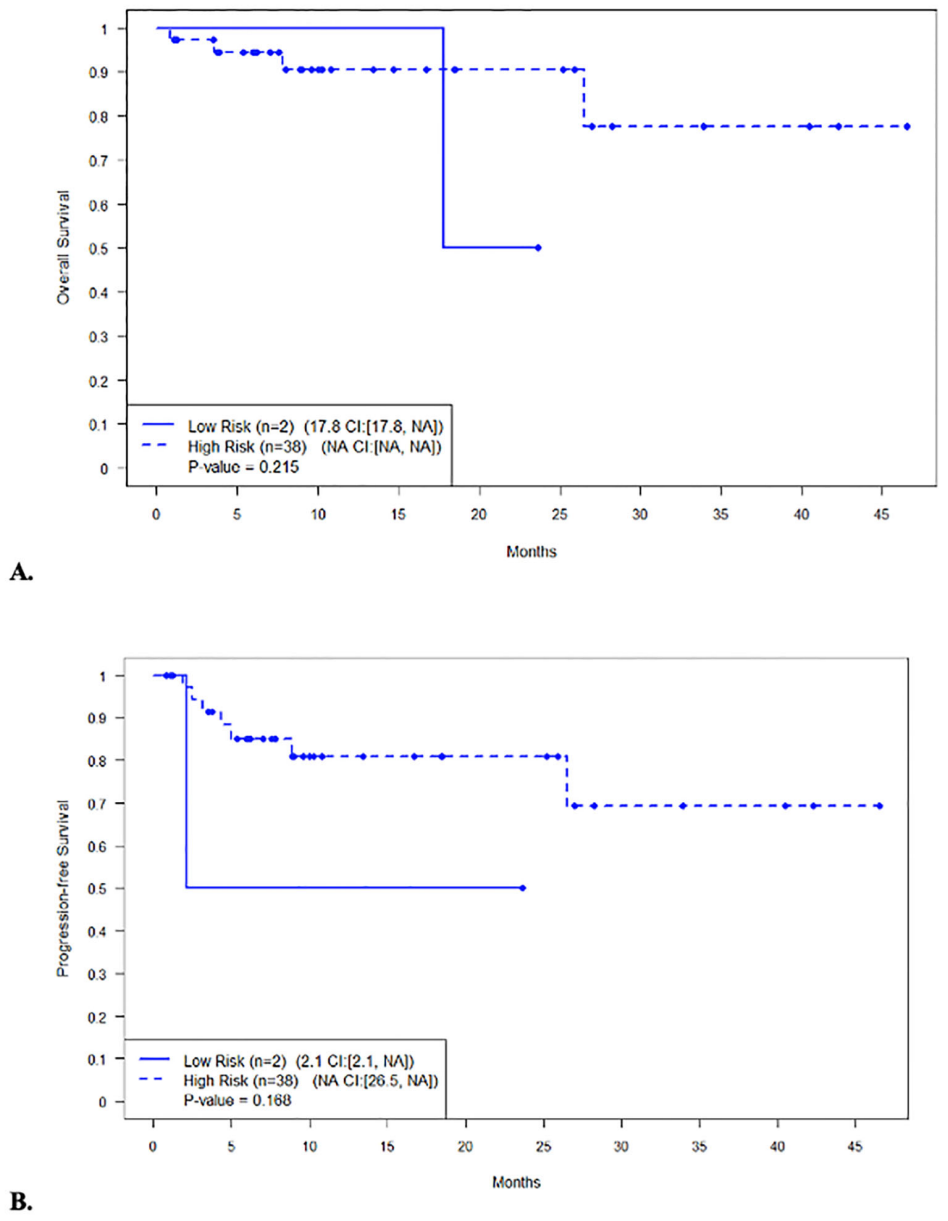
Survival in patients with disease encompassable in a standard RT plan (less than 5 lesions) with high-risk vs not high-risk features. **(A)** Overall survival. **(B)** Progression-free survival.

**Figure 3:**
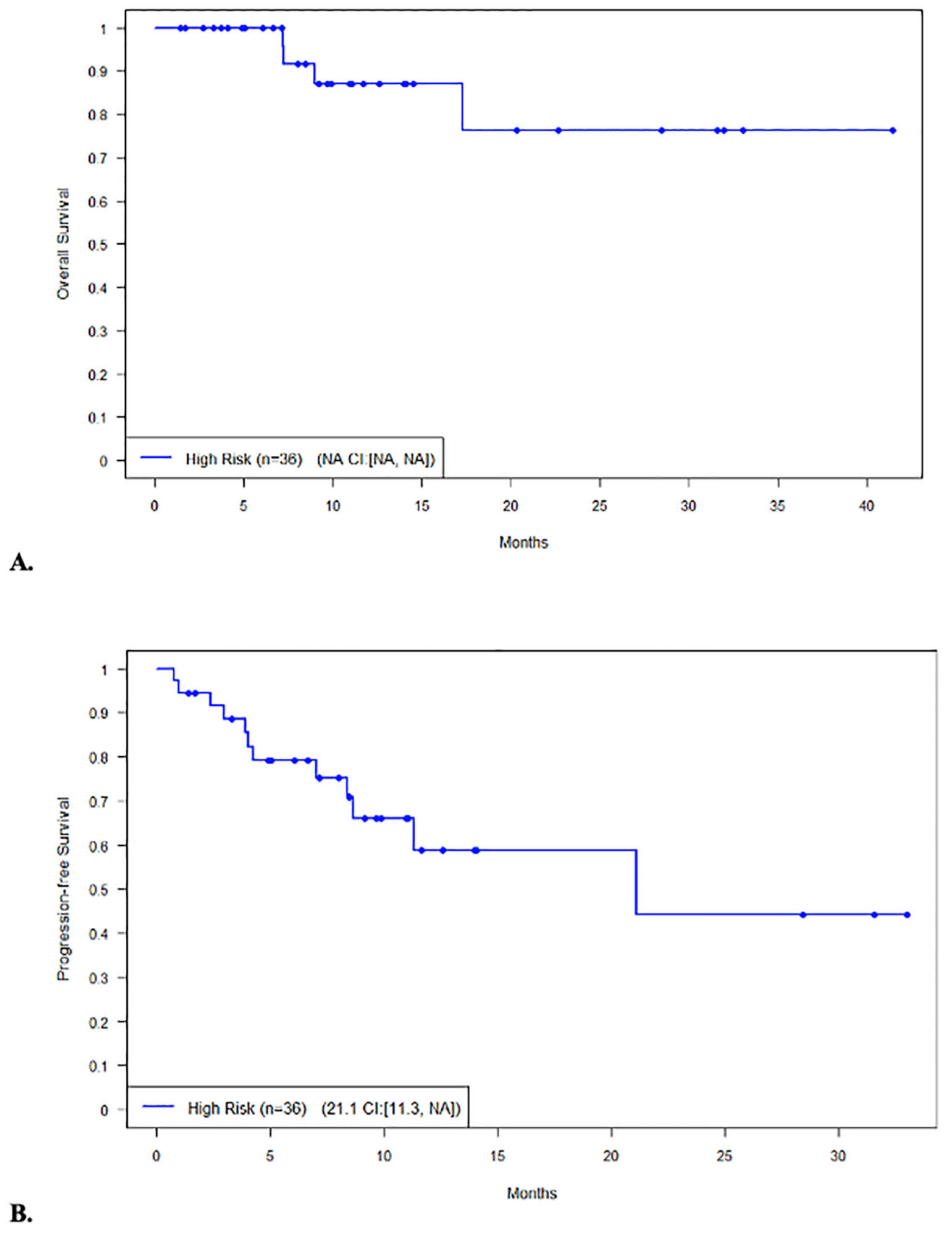
Survival in patients (n=36) not eligible for bridging RT (≥5 lesions) with high-risk features. **(A)** Overall survival. **(B)** Progression-free survival.

The median PFS and OS for all patients were not reached (NR), respectively (**Figures 1A, B**). In patients with disease encompassable in a standard RT plan, the median OS comparing those with high-risk (*n* = 38) and low-risk (*n* = 2) features was not reached (95% CI NR, NR) vs. 17.8 months (95% CI 17.8, NR, *p* = 0.215), respectively (**Figure 2A**). In addition, the median PFS comparing those with high-risk (*n* = 38) and low-risk (*n* = 2) features was not reached (95% CI 26.5, NR) vs. 2.1 months (95% CI 2.1, NR, *p* = 0.168), respectively (**Figure 2B**). In patients with high-risk features but with more than five lesions on pre-apheresis studies, the median OS was not reached (95% CI NR, NR) and median PFS was 21.1 months (95% CI 11.3, NR) (**Figures 3A, B**).

Of the 40 patients who met our eligibility criteria for bridging RT, 9 patients received any bridging therapy prior to CD19 CART, including 7 patients who received bridging systemic chemotherapy (ST) and 2 who received bridging RT. Of the patients who received bridging ST with response data available (*n* = 6), the ORR at 3 months was 66% (33% CR; 33% PR). One patient who received bridging RT had persistent disease after three cycles of chemoimmunotherapy with R-CHOP and R-hyperCVAD (hyperfractionated cyclophosphamide, vincristine, doxorubicin, and dexamethasone). This patient went on to receive bridging RT at a total dose of 4,140 cGy (180 cGy in 23 fractions) to a single site of active disease in the right occipital scalp before CD19 CART and remained in continued CR at 23 months of follow-up. Another patient with non-double hit, germinal center B-cell-like (GCB) DLBCL received six cycles of R-CHOP and achieved CR but relapsed less than 1 year later. This patient went on to receive R-ICE (ifosfamide, cyclophosphamide, and etoposide) for two cycles with PD followed by bridging RT at a total dose of 3,000 cGy (300 cGy in 10 fractions) to a single site of active disease in the left shoulder before CD19 CART. The patient experienced relapse in the left scapula (outside the radiation field) 6 months after CD19 CART.

## 4 Discussion

CD19 CART offers a promising treatment option in patients with R/R NHL as nearly 50% to 70% of patients will be alive at 1 year after treatment (2). Nevertheless, approximately half of patients experience relapse after CD19 CART with an associated life expectancy after relapse of less than 1 year (2). Prior studies described disease features at the time of CART that increase the likelihood of early relapse after CART in patients with R/R NHL (6, 7), particularly bulky or necrotic disease found on pre-treatment staging studies. Here, we investigated the proportion of patients with R/R NHL at high risk of early progression after CD19 CART who presented with disease potentially encompassable in a standard radiation plan feasible using current technology. In our cohort, we found that nearly half (49%) of patients who underwent CD19 CART would have been eligible for bridging RT based on their disease burden of <5 lesions on pre-apheresis imaging studies. Within this subset, the majority (38/40, 95%) of patients met high-risk criteria for early progression after CART. These patients potentially would have benefitted from bridging RT, which, in addition to its palliative effects that may improve survival, is thought to have a role in potentiating the effects of CART to improve response rates and prevent early disease relapse (18).

Radiotherapy is traditionally used in the management of NHL for the purpose of tumor debulking or symptom palliation as NHL is a radiosensitive disease (19). In the setting of CART, tumor debulking is a potentially beneficial strategy to employ as decreased tumor burden is associated with improved response rates and decreased toxicity (20). In the ZUMA-1 trial, lower tumor burden was associated with an increased ORR in patients who received axicabtagene ciloleucel, as well as associated with lower rates of grade 3 or higher cytokine release syndrome (CRS) and neurologic events. In addition, a study at the Moffitt Cancer Center in patients with large B-cell lymphoma who received axicabtagene ciloleucel found that elevated tumor burden was associated with significantly shorter PFS and OS (21). These studies suggest that pre-treatment tumor debulking may improve CART outcomes, and preliminary data evaluating this strategy support this notion (22). Qu et al. described a phase 2 study involving bridging RT in six patients with diffuse large B-cell lymphoma (DLBCL) with bulky disease and found that these patients achieved a higher ORR (100% vs. 25%) than those who received bridging ST, and lower CRS (0% vs. 100%) and neurologic events (75% vs. 90%), respectively (23). Sim et al. described a retrospective study of 12 patients with bulky NHL who received bridging RT and reported an ORR of 81.8% (CR 45.5%), with only one and three events of severe CRS and neurologic events, respectively (9).

In addition to the potential benefit of tumor debulking, radiotherapy has been shown to enhance the treatment of chemo-refractory lymphomas through prevention of locoregional relapse and improved response (24). A study comparing bridging RT, bridging ST, and no bridging therapy (NBT) showed that patients with DLBCL who underwent bridging RT had a longer PFS than those who underwent bridging ST. Patients who received bridging RT also had higher rates of CR than those in the bridging ST and NBT groups. The better response observed with bridging RT could be explained by differences in patients’ tumor burdens, as patients who undergo bridging ST may have a higher tumor burden or less localized disease compared to patients who underwent bridging RT (10). Additionally, patients in the bridging RT cohort had an average of two prior lines of therapy before CART whereas patients who received bridging ST had an average of three prior lines of therapy, suggesting possible differences in disease characteristics. However, the proportion of patients with bulky disease, elevated LDH, and other advanced disease characteristics was not statistically different between those who received bridging RT compared to ST. In our study, there were 9 patients (31%) with a PR or less (PR 7%; SD 14%; PD 10%), including 7 patients who received bridging ST. Bridging ST is predictive of poor response to CART, as these patients tend to have higher relapse rates likely due to a more aggressive disease biology (25). This is consistent with our study where the ORR at 3 months was 57% (4/9; 2 CR; 2 PR) in patients who received bridging ST. Bridging RT may have played a role in improving response or providing locoregional control of disease in this setting.

A hypothesis under active study is that bridging RT with defined fractionation and total dose administered may modulate the TME and immune response to CART (13, 14, 26). Dutt et al. illustrated this concept of non-standard RT in a murine model of human NHL using a conventional RT dose (3 Gy in 10 daily fractions) administered over 12 days compared to the same total dose (3 Gy in 10 fractions) administered over 4 days (26). The accelerated RT dose was associated with more complete and durable remissions, and the majority of these mice were resistant to rechallenge with lymphoma cells demonstrating the induction of memory antitumor immunity. In a murine model of glioblastoma, Weiss et al. demonstrated that the cell infusion from natural killer cell-derived CART would cure 22% of mice treated (27). The addition of a single, 400-cGy fraction of cranial RT achieved improved trafficking of CART into the tumor site and improved survival. In addition, the potential of the TME to suppress responses to immunotherapies, such as checkpoint inhibitors and CART, may be reduced through the addition of RT. Buchwald et al. concluded that, at an optimal RT dose and fractionation (8 and 10 Gy per fraction in one to three fractions) in combination with checkpoint inhibitors, there appears to be an effective antitumor response and abscopal effect in preclinical models (14).

This study has several major limitations. To begin, the single-center design is associated with inherent limitations, such as a small sample size and different NHL subtypes that reduce the generalizability of our results. Given the retrospective nature of our study, it is important to acknowledge that delineation of our patient population may be subject to selection bias or missing data from patient charts. Finally, our data show that a large proportion of patients could receive bridging RT, but the potential of an immunological benefit of RT will require prospective studies of this modality with appropriate laboratory correlates.

In conclusion, effective strategies for preventing relapse among patients who receive CD19 CART are needed. Our analysis showed that approximately 47% (38/81) of patients were classified as presenting with disease encompassable in a standard radiation plan and could be referred for bridging RT as palliative therapy or in a study of the immunomodulatory effects of RT. Future studies are needed to determine the role of bridging RT before CD19 CART as a strategy to improve outcomes and prevent early relapse in patients with R/R B-cell NHL at high risk of progression.

## Data Availability

The complete datasets used and/or analyzed during this study are available from the corresponding author upon request. Requests can be made through the corresponding author or directly to representatives of Hackensack Meridian Health (Adnan Danish; Email: Adnan.Danish@hmhn.org).
