# Supplementary figures and images for: Prevalence of non-Hodgkin lymphoma patients at high-risk of failure after CAR T-cell therapy eligible for bridging radiation therapy
Source: Front Oncol. 2024 Aug 19;14:1425506. doi: 10.3389/fonc.2024.1425506 (PMC11369895; doi:10.3389/fonc.2024.1425506)

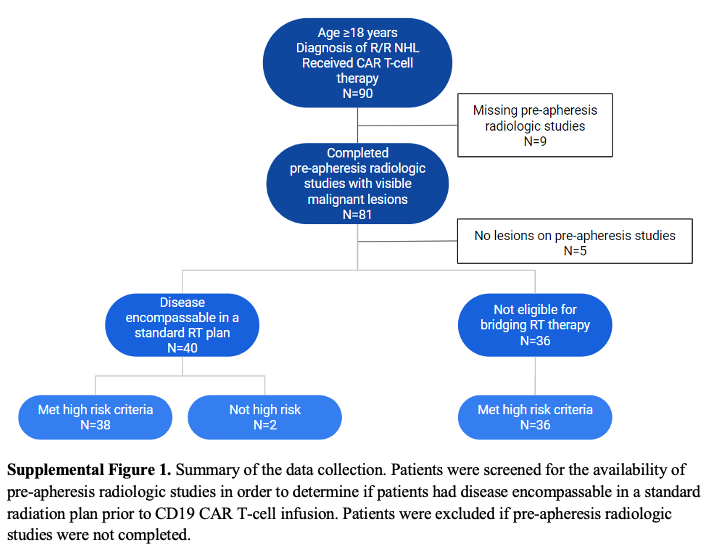

Supplement: Supplementary file 1 [file Image1.tiff]
